# Evolutive assessment of endothelial function in metabolic control and microvascular complications in type 1 diabetes mellitus patients

**DOI:** 10.1186/1758-5996-7-S1-A37

**Published:** 2015-11-11

**Authors:** Winston Boff, Antonio Marcos Vargas da Silva, Marcia Puñales, Gislaine Vissoky Cé, Marcello Casaccia Bertoluci

**Affiliations:** 1 Hospital da Criança Conceiçao e Instituto da Criança Com Diabetes-GHC, Porto Alegre, Brazil

## Background

Long-term type 1 diabetes (T1D) is associated to microvascular complications. In a previous study of our group, we observed that endothelial dysfunction (ED) was present in half of adolescents under 5 yrs. of T1D duration, even before the onset of microvascular disease. Although ED is associated to vascular complications, the natural history and risk of developing these complications is not yet understood.

## Objective

The aim of the study was to determine the potential evolution of ED overtime and if its determination could be predictive for albuminuria in T1D patients.

## Materials and methods

Observational cohort study with 7 yrs. of follow up, including 40 T1D patients, mean age of 22.6±4.2 yrs. The assessment of endothelial function and vascular stiffness was performed respectively through flow-mediated dilation (FMD%) in brachial artery and dilation mediated by nitroglycerin (NTG%). ED was defined by% of dilation inferior to 8%. HbA1c was measured at 3 months intervals over 7 yrs. Data were analyzed comparatively between 2007 and 2014 for%ED (2007: ED1 and 2014: ED2) and%NTG (NTG1 and NTG2), using t-test for dependent samples and McNemar paired test to comparison.

## Results

Data were available of 40 T1D patients, mean follow up of 7 yrs. Mean and SD for ED and NTG were respectively: ED1: 9.6±6.8 vs ED2: 6.9±4.9%, p=0.0032 and NTG1: 22.9±9.8 vs NTG2: 16.8±4.5%, p=0.000. Of 40 patients, 55.0% did not have ED in 2007, and 25.0% of them developed new ED in 7 yrs. Of the patients who had ED in 2007, 27.5% remained in ED in 2014 while 17.5% ED patients in 2007 reverted to normal in 2014. A total of 45% of the patients had ED in 2014, being independent of HbA1c. HbA1c in those with and without ED in 2014 was respectively: 8.8±1.7 and 8.6±1.7% (0.646). Interestingly, 47.5% patients improved ED to normality, despite a slight increase in HbA1c of 0.06±1.9%. The presence of albuminuria was not associated with ED, NTG or HbA1c. The UAE was: 28.7±62.7 vs 25.8±54.5mg/dL in ED and Non-ED, respectively (p=0.784) and 29.6±63 vs 26.2±55mg/dL in NTG or Non-NTG (p=0,761).

## Conclusion

The resuts showed that in most T1D both ED and vascular stiffness deteriorate significantly after 7 yrs., being reversible in some patients. Our data suggest that T1D duration is the main predictor of deterioration of ED, independently HbA1c or albumiruria. Longer studies are needed to define whether ED can be used as a marker of future atherosclerosis in T1D.

